# Effective health management strategies for patients undergoing valve replacement: a bibliometric analysis of the current research status and future directions

**DOI:** 10.3389/fcvm.2024.1352437

**Published:** 2024-02-26

**Authors:** Xiaohui Wang, Ying Wu, Ming Li, Jing Wen, Lijuan Liu, Wenzhuo Huang, Qianqian He, Yongzhen Liao, Li Cong

**Affiliations:** ^1^ School of Medicine, Hunan Normal University, Changsha, Hunan, China; ^2^ Trauma Center, Zhu Zhou Hospital Affiliated to Xiangya School of Medicine, Central South University, Zhuzhou, Hunan, China; ^3^ Nursing Department, Hunan Cancer Hospital, Changsha, Hunan, China; ^4^ Teaching and Research Section of Clinical Nursing, Xiangya Hospital of Central South University, Changsha, Hunan, China; ^5^ Shunde Polytechnic, Foshan, Guangdong, China

**Keywords:** valve replacement, management, CiteSpace, VOSviewer, bibliometrics, visualization

## Abstract

**Background:**

Valvular heart disease is a major health concern worldwide. The effective management of patients undergoing valve replacement determines their prognosis. Bibliometric analysis of studies on managing patients with artificial heart valves has not been previously performed.

**Methods:**

This study analyzed 2,771 publications related to patient management after valve replacement published in the Web of Science Core Collection database between January 1, 2013, and December 31, 2022. Bibliometric analysis was performed using CiteSpace and VOSviewer considering countries, institutions, authors, journals, references, and keywords.

**Results:**

The countries with the most significant contributions in this field were the United States of America (USA), Germany, and Italy. Leon MB from Columbia University, USA was the most influential author. Transcatheter aortic valve replacement was a current research hotspot, while anticoagulation management was a key area of interest. Combining anticoagulation therapy with internet-linked tools and portable health devices may offer new research avenues. Frailty assessment and intervention were potential future research areas.

**Conclusions:**

This bibliometric analysis provides clinicians and researchers with useful insights for developing novel ideas and directions to manage the health of patients undergoing valve replacement.

## 1 Introduction

Valvular heart disease (VHD), a prevalent cardiovascular condition, is characterized by the narrowing or regurgitating of one or more valves, including aortic, mitral, tricuspid, and pulmonary valves (1). The incidence rate of VHD in the general population is more than 2%. Globally, VHD is a major etiological factor for cardiovascular morbidity and mortality (2, 3). The prevalence rates of VHD are increasing with population aging. Additionally, the prevalence rate of VHD in individuals aged ≥65 years is estimated to be 11.3% and may double by the end of 2050 (4, 5). The burden of managing VHD is rapidly increasing owing to the global aging population and the utilization of healthcare resources for patients with VHD (6). VHD can be classified into different types depending on various factors, including genetics, age, gender, and environment. Degenerative or functional VHD is prevalent in high-income countries, while rheumatic heart disease is prevalent in middle and low-income countries (3, 7). Valve replacement is the primary treatment for VHD. However, patients are at high risk of heart failure and rehospitalization after valve replacement. Valve repair can avoid the burden of lifelong anticoagulant treatment for patients but increases the risk of reoperation (8). Different postoperative management strategies are associated with different risk levels and complications, differentially affecting the quality of life of patients (9). Therefore, there is a need to develop appropriate customized strategies for each patient undergoing valve replacement to ensure safety and improve clinical outcomes.

The prognosis of patients undergoing valve replacement is dependent on several factors, including surgical choice, medication compliance, implant performance, the severity of complications, and quality of life (10, 11). To prolong survival duration and improve rehabilitation outcomes, patients must regularly undergo blood coagulation tests, cardiac function assessments, anticoagulant therapy, complication nursing, and other measures (12). The management of patients undergoing valve replacement includes hospital-based and self-management approaches, which aim to achieve optimal long-term outcomes through periodic monitoring and timely intervention (13). Various intelligent rehabilitation health management strategies, such as wearable monitoring devices, self-management applications, and Internet Plus, have emerged in recent years in medical care (14–16). Thus, gaining insight into the trends and identifying future opportunities in managing patients undergoing valve replacement may promote mobile intelligent health management, provide an integrated and innovative service mode, and achieve optimal health management.

Bibliometrics is an auxiliary research method proposed in 1969 to analyze the current status and track development trends in a particular field (17). This approach involves analyzing the literature, cooperative relationships, international influence, and hot research topics to obtain objective evidence and offer valuable insights for clinical decision-making. Bibliometrics has widespread applications in several disciplines, such as informatics, economics, and medicine. This method has been used in the cardiovascular field to study cardiac regeneration, exosomes in cardiovascular disease, and digital technology applications in cardiology (18–20). Bibliometric analysis of studies related to managing patients undergoing valve replacement will comprehensively reveal the structure and development of the management strategies, providing intuitive information and potential avenues for future research. However, bibliometric analysis of studies on the health management strategy for patients undergoing valve replacement has not been previously performed.

This study aimed to perform a statistical analysis of publications related to patient health management after valve replacement published in the Web of Science Core Collection database over the past decade. The quality and influence of the literature were systematically described by quantifying and analyzing the basic information of countries, institutions, authors, and journals. The collection of references and keywords enabled the identification of popular research objects and the prediction of the hotspots and frontiers in the management of patients undergoing valve replacement. Additionally, VOSviewer and CiteSpace were used to generate knowledge maps, which offer valuable insights to guide future research endeavors in managing patients undergoing valve replacement.

## 2 Methods

### 2.1 Data source and search strategy

The Web of Science Core Collection (WOSCC) database, an authoritative source for indexing high-quality literature, is frequently selected for bibliometric analyses in various fields, including natural sciences, arts and humanities, engineering and technology, social sciences, and biomedical sciences (21, 22). To improve data availability, the WOSCC database was queried for literature related to the health management of patients undergoing valve replacement. The data search strategies were as follows: {[TI = (“valve replace*” or “valve implant*”)] OR AK = [“valve replace*” or “valve implant*”]} AND TS = (monitor or surveillance or manage or management or administer). The period selected for literature review was between January 1, 2013, and December 31, 2022. Two independent researchers (XHW and YW) completed the literature search. To prevent errors resulting from database updates, the investigation was concluded on January 1, 2023. The relevant information, such as title, abstract, keywords, references, authorship, and article sources, was saved in “txt” format for further analysis.

### 2.2 Data collection and analysis

**Figure 1:**
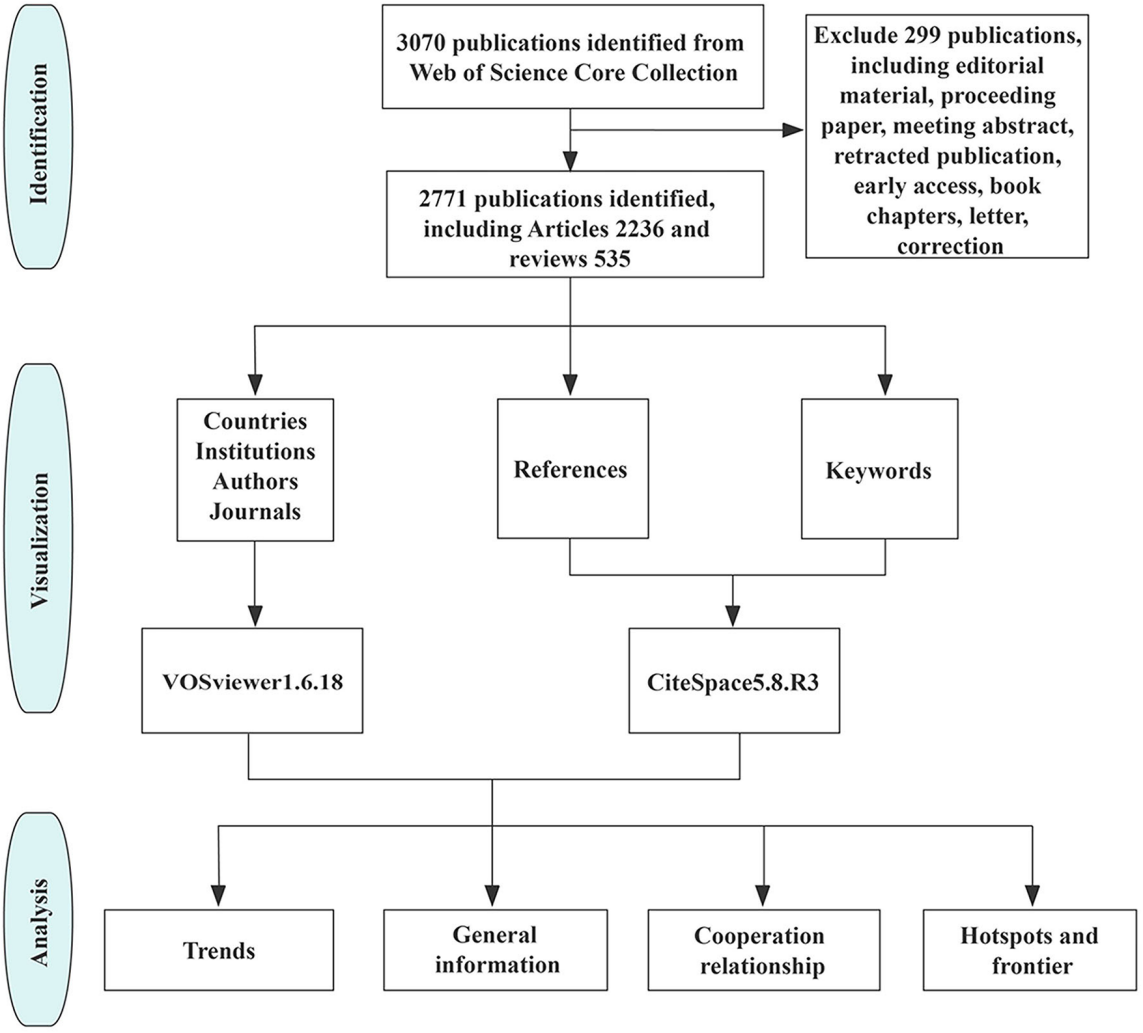
The flowchart of the literature identification, visualization, and analysis.

The records collected from WOSCC were exported to plain text files and then imported into VOSviewer 1.6.18 and CiteSpace 5.8.R3 for bibliometric analysis and visualization. The flowchart is shown in Figure 1.

VOSviewer, a widely used tool for bibliometric analysis, can provide practical visualization effects and highlight connections between materials (23, 24). This software has a powerful bibliometric map function that generates networks of countries, keywords, authors, and other relevant factors (25). In this study, VOSviewer was used to visualize networks of countries, institutions, authors, co-cited authors, and co-cited journals to show general information and global research progress on managing the health of patients undergoing valve replacement.

CiteSpace, which was designed by Professor Chaomei Chen at Drexel University, is a Java application that enables the generation of maps demonstrating the evolution of data from various perspectives (26). In particular, CiteSpace can identify collaborative networks among countries, references, and authors and identify the key turning points and the most widely cited bursts in a specific field (27). In this study, CiteSpace was used to exhibit reference bursts, keyword bursts, and a timeline viewer of keywords to explore the progression of research hotspots and predict future developments in the health management of patients undergoing valve replacement.

## 3 Results

### 3.1 Analysis of the trend of publication in the past decade

**Figure 2:**
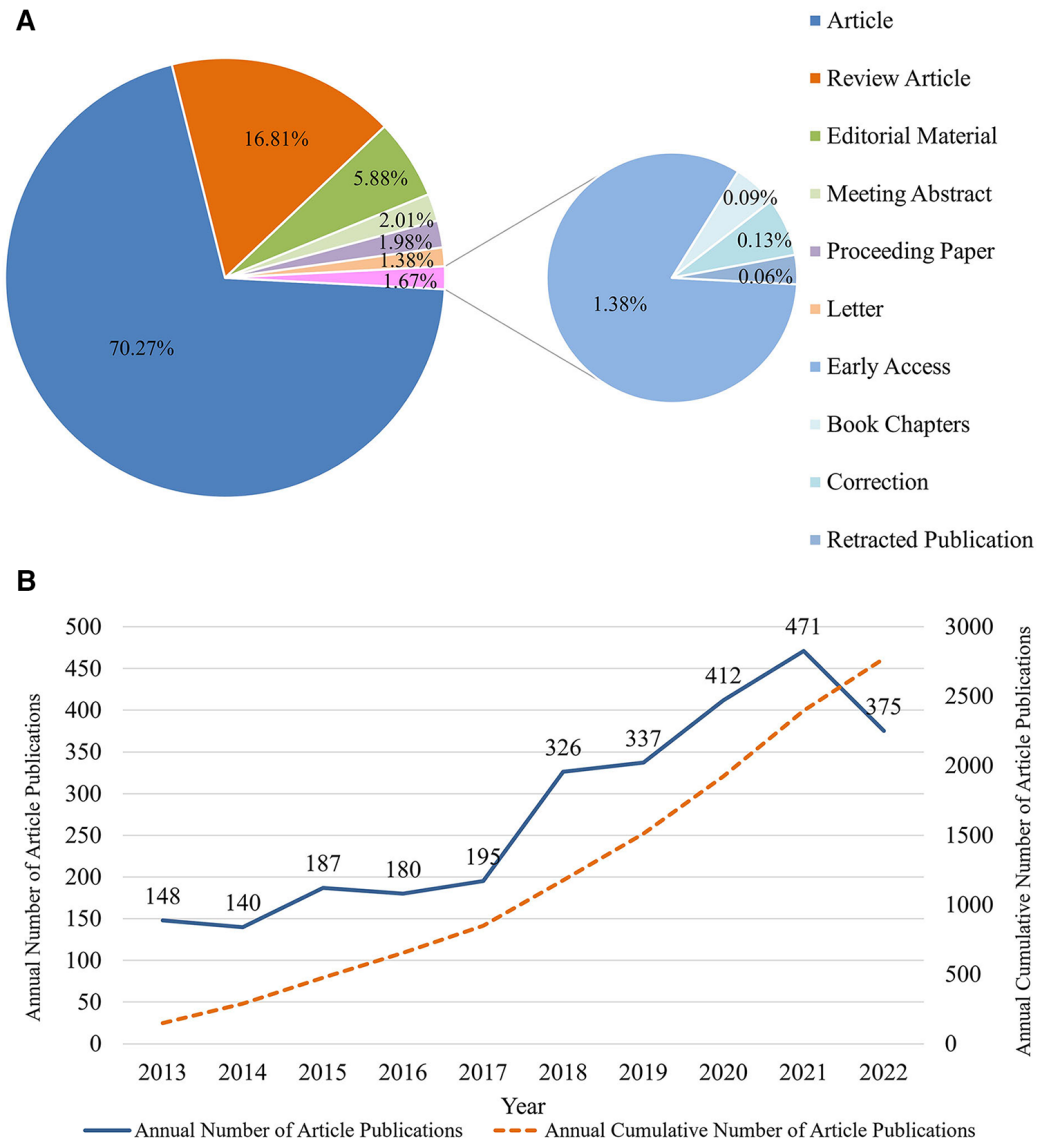
Document types and publications trend for the health management of valve replacement patients. (**A**) Document types. Description: Different colors correspond to different literature types, and the percentages of different types are annotated in the corresponding color areas. (**B**) Annual and cumulative publications trend. Description: The blue line represents the annual publication trend; the orange line indicates the cumulative publications trend. The number of documents published per year is marked above the blue line.

Several types of manuscripts, such as articles, reviews, editorial materials, meeting abstracts, proceeding papers, letters, early accesses, book chapters, corrections, and retracted publications were available in the literature (Figure 2A). This study focused on articles and reviews. Based on the search strategy, 2,771 publications comprising 2,236 articles and 535 reviews were retrieved. Additionally, the number of annual and cumulative publications broadly exhibited an upward trend (Figure 2B). From 2013 to 2017, the growth rate of publication volume was low (an average of 170 publications per year). Since 2018, the growth rate of publication volume rapidly increased (>300 publications per year). In 2021, 471 papers were published, indicating that the health management of patients undergoing valve replacement piqued the interest of the scientific community. The number of publications in 2022 was lower than that in 2021. However, the number of publications in 2022 was as high as 375, indicating sustained research interest in this area.

### 3.2 Contributions of country/region and institution

**Figure 3:**
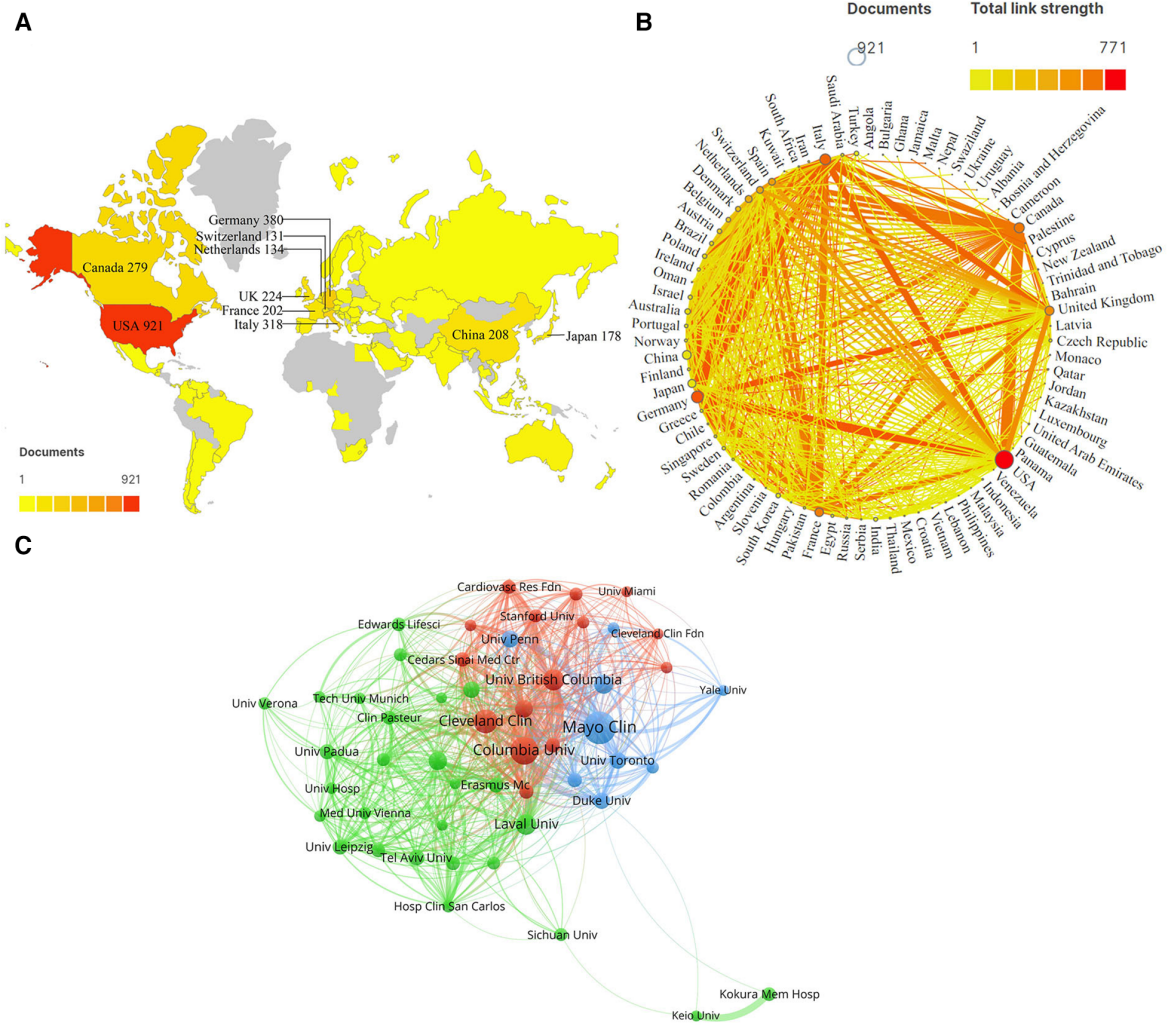
Collaboration of countries and institutions associated with managing patients undergoing valve replacement. (**A**) Geographical distribution of publications. Description: The labels refer to ten countries with high publication yields and their output; the shade of color matches the number of documents. (**B**) International cooperation among countries. Description: Each node denotes one country. The curves correlate to collaboration between countries, and the wider the ribbon, the closer the cooperation. (**C**) Collaboration networks among institutions. Description: Node size depends on the number of institutional publications; node colors represent different clusters in which the representative organizations can be found. The line shows the collaboration between institutions; the thicker the line, the tighter the cooperation.

Between 2013 and 2022, 91 countries (37 high-income and 54 low-income and middle-income countries) contributed to the literature on the health management of patients undergoing valve replacement. Only seven low-income and middle-income countries were among the top 30 contributors to publication volume. This indicated that the research in this field in high-income countries was higher than that in low-income and middle-income countries, which may be related to publication bias. The most productive country was the United States of America (USA) (*N* = 921; 33.24%), followed by Germany (*N* = 380; 13.71%) and Italy (*N* = 318; 11.48%) (Table 1; Figure 3A). The USA, Germany, and Italy accounted for more than half of all publications in this field. Collaborative efforts between nations are critical for scientific research and medical advancement. As shown in Figure 3B, the USA had the highest total link strength and cooperation relationship, especially with Canada, followed by Germany, Italy, the United Kingdom, and France. Although China and Japan were ranked among the top 10 countries contributing to publication volume, their cooperation intensity with other nations was inadequate.

**Table 1:** Top 10 countries/regions and institutions related to the health management of patients with valve replacement.

Rank	Country/Region	Count/Proportion	Total link strength	Institution	Count/Proportion	Total link strength
1	USA	921 (33.24%)	771	Mayo Clin (USA)	112 (4.93%)	591
2	Germany	380 (13.71%)	678	Columbia Univ (USA)	88 (3.87%)	956
3	Italy	318 (11.48%)	658	Cleveland Clin (USA)	70 (3.08%)	628
4	Canada	279 (10.07%)	605	Univ British Columbia (Canada)	58 (2.55%)	420
5	United Kingdom	224 (8.08%)	517	Laval Univ (Canada)	54 (2.38%)	593
6	China	208 (7.51%)	70	Harvard Med Sch (USA)	49 (2.16%)	236
7	France	202 (7.29%)	592	Univ Catania (Italy)	46 (2.03%)	583
8	Japan	178 (6.42%)	55	Emory Univ (USA)	43 (1.89%)	566
9	Netherlands	134 (4.84%)	389	Univ Penn (USA)	40 (1.76%)	190
10	Switzerland	131 (4.73%)	429	Univ Toronto (Canada)	39 (1.72%)	291

Additionally, 3,601 institutions were involved in this study. Among the top 10 organizations ranked according to publication volume, six were in the USA, three were in Canada, and one was in Italy (Table 1). The Mayo Clinic was the most prolific research organization performing studies on the health management of patients undergoing valve replacement (*N* = 112, 4.93%), followed by Columbia University (*N* = 88, 3.87%) and Cleveland Clinic (*N* = 70, 3.08%). The increased academic exchanges have contributed to inter-institutional collaborations. This study provided an illustrative network visualization among these organizations (Table 1; Figure 3C). Columbia University exhibited the highest total link strength (*N* = 956) and the closest cooperative relationship with the Cardiovascular Research Foundation, while the Mayo Clinic exhibited the most immediate cooperation with the University of Pennsylvania.

### 3.3 Analysis of authors and co-cited authors

**Figure 4:**
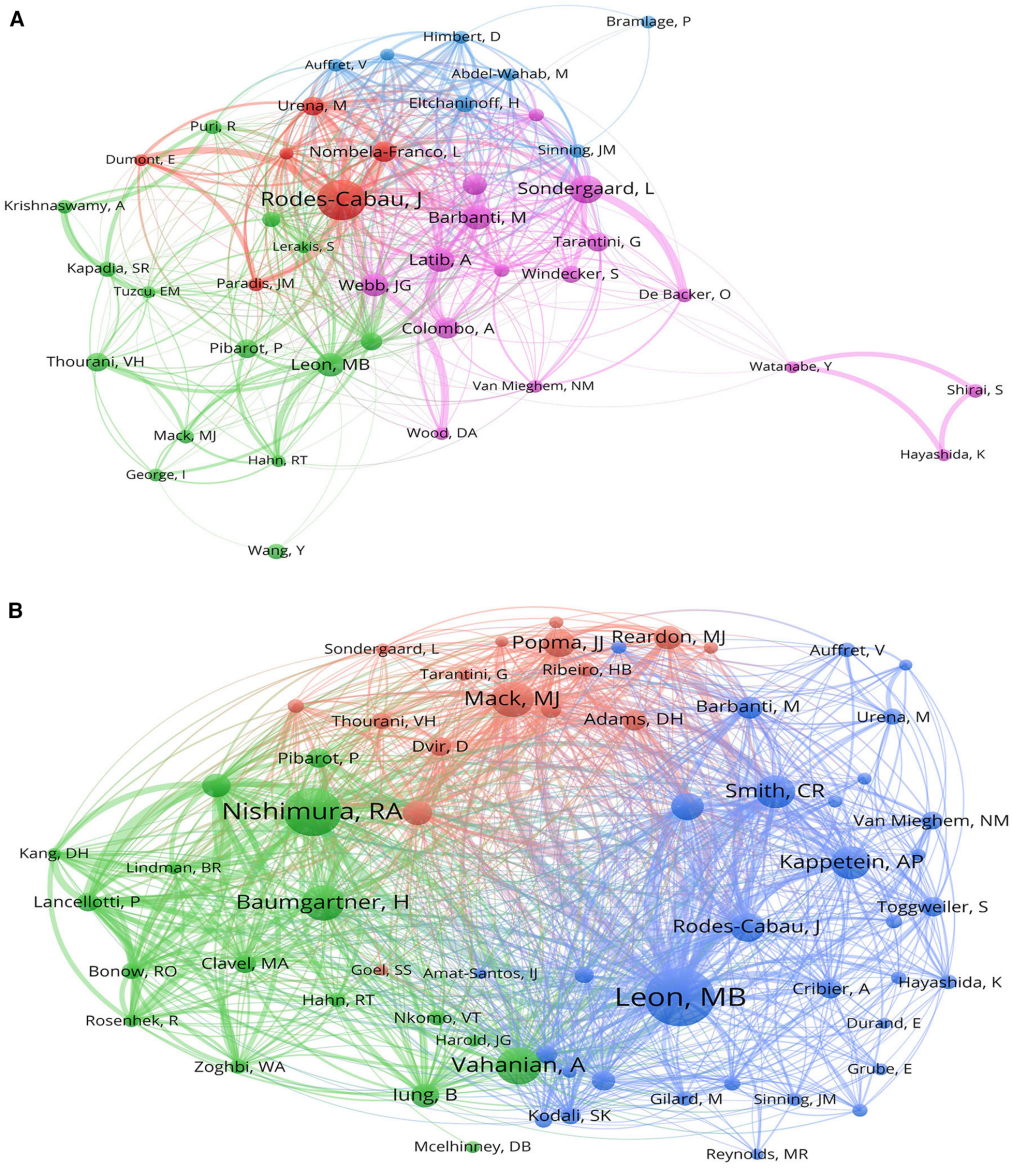
VOSviewer network visualization maps of authors and co-cited authors. (**A**) Cooperation network of authors. Description: Node size is affected by the author’s publication volume, and node colors represent different clusters in which the authors’ research groups can be found. The line connecting two nodes is related to the strength of the authorship collaboration, and the thicker the line, the tighter the cooperation. (**B**) The network visualization map of co-cited authors. Description: Node size is determined by the co-citation frequency of authors, and node colors represent different clusters in which the authoritative authors can be found. The line between the nodes means a co-citation relationship; the broader the line, the more similar the research areas of the two co-cited authors.

In the last decade, 14,197 authors contributed to studies on the health management of patients undergoing valve replacement. The most productive author was Rodes-Cabau J (Quebec Heart & Lung Institute, Canada) with 75 publications, followed by Sondergaard L (Glenfield Hospital, Denmark) and Barbanti M (University of Catania, Italy) with 50 and 44 publications, respectively (Table 2). Long-term collaboration between researchers enhances the influence of authors and facilitates the development of patient health management strategies. Figure 4A shows the collaboration network among authors. Rodes-Cabau J conducted frequent academic collaborations with Nombela-Franco L, Urena M, and Dumont E, who were affiliated with the Quebec Heart & Lung Institute. Co-citation analysis enables the identification of authors who had the highest contributions to studies on the health management of patients undergoing valve replacement (Figure 4B). Leon MB was the most frequently co-cited author despite ranking fifth in publication volume, suggesting that the results from this team have gained high recognition and reference value. Moreover, Leon MB and Smith CR exhibited the closest co-citation relationship, indicating that they share similar research topics and are often cited together by other authors.

**Table 2:** Top 10 authors and co-cited authors related to the health management of patients with valve replacement.

Rank	Author	Country	Count	Institution	Co-cited author	Country	Citation	Institution
1	Rodes-Cabau J	Canada	75	Quebec Heart & Lung Institute	Leon MB	USA	1,228	Columbia University
2	Sondergaard L	Denmark	50	Glenfield Hospital	Nishimura RA	USA	940	Mayo Clinic
3	Barbanti M	Italy	44	University of Catania	Vahanian A	France	657	UDICE-French Research Universities
4	Latib A	Italy	42	IRCCS San Raffaele Scientific Institute	Baumgartner H	Germany	615	University of Munster
5	Leon MB	USA	42	Columbia Intervent Cardiovasc Care	Mack MJ	USA	613	Baylor University Medical Center
6	Webb JG	Canada	40	University of British Columbia	Smith CR	USA	558	New York-Presbyterian Hospital
7	Colombo A	Italy	38	IRCCS Humanitas Research Hospital	Kappetein AP	Netherlands	549	Erasmus University Rotterdam
8	Nombela-Franco L	Spain	36	Quebec Heart & Lung Institute	Rodes-Cabau J	Canada	460	Quebec Heart & Lung Institute
9	Tamburino C	Italy	35	University of Catania	Genereux P	USA	426	Atlantic Health System
10	Pibarot P	Canada	33	Quebec Heart & Lung Institute	Popma JJ	USA	416	Harvard University

### 3.4 Analysis of journals and co-cited journals

**Figure 5:**
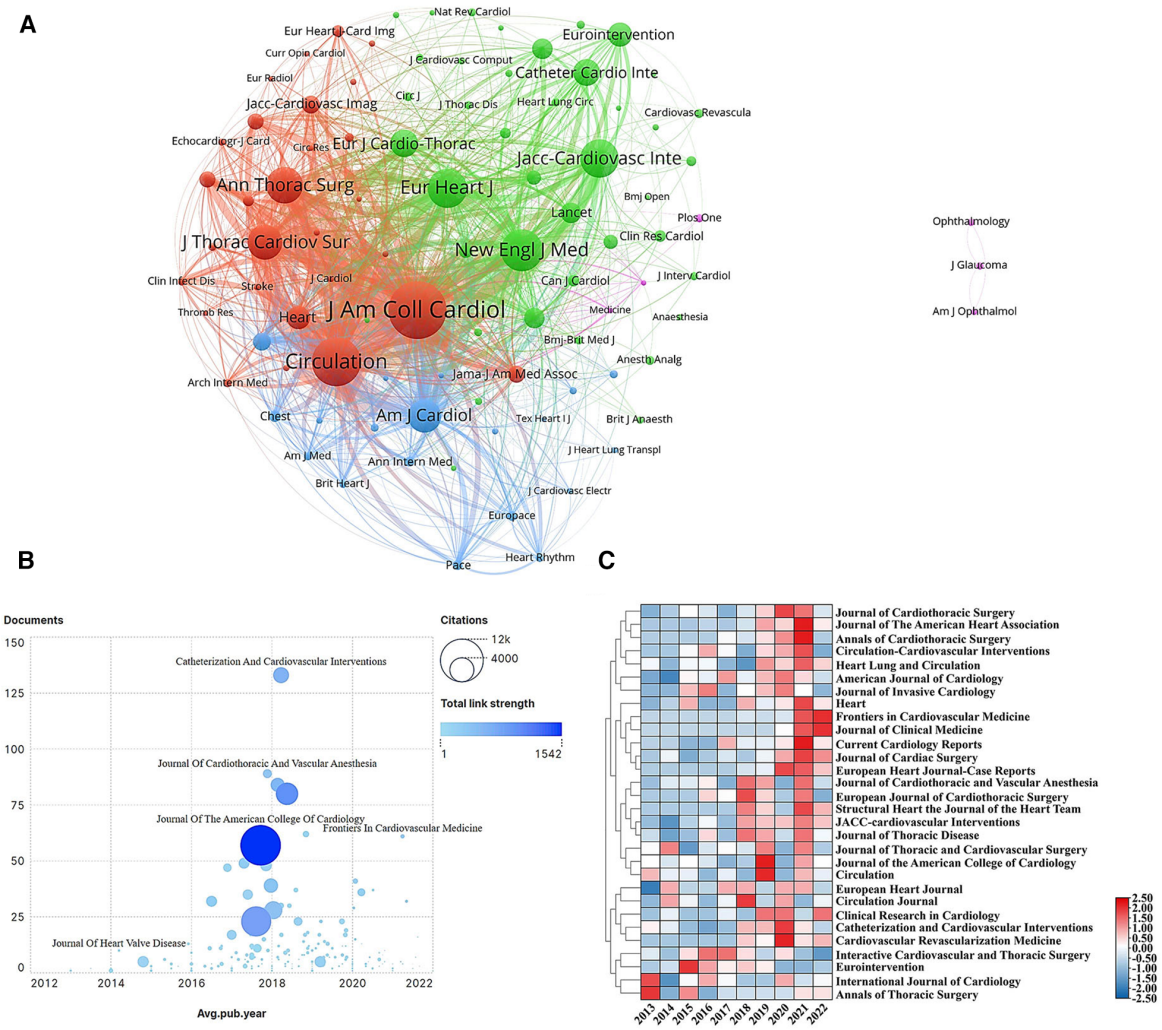
VOSviewer network visualization map of co-cited journals, and journals heat map. (**A**) Network visualization of co-cited journals. Description: Each node stands for a co-cited journal, and node colors represent different clusters in which popular journals can be found. The line with color corresponds to the co-citation relation between journals. (**B**) Journal documents, citation frequency, and total link strength over time. Description: Each node indicates one journal. Larger nodes mean higher citation frequencies; the darker the color, the higher the strength of links. The ordinate denotes the journal publication volume. The abscissa represents the average publication year of the journal, and the smaller the year, the earlier the journal published the literature. (**C**) Heat map of journals. Description: The numbers on the right are normalized and colored. Each journal’s publication trends over the years can be analyzed individually; a higher value means a redder color, indicating a better trend in journal publication.

In total, 2,771 articles related to the health management of patients undergoing valve replacement were distributed across 525 journals. Table 3 displays the top 10 journals sorted according to publication volume. Impact factor (IF) and journal citation reports were also presented to weigh the scientific research quality of journals. The three most common journals in which the articles were published were Catheterization and Cardiovascular Interventions (133 papers, IF: 2.585), Journal of Cardiothoracic and Vascular Anesthesia (89 papers, IF: 2.894), and American Journal of Cardiology (84 papers, IF: 3.133). In addition to the number of published articles, the influence of the journal is dependent on the frequency of citations. Journal of the American College of Cardiology, Circulation, and New England Journal of Medicine were among the most frequently co-cited journals (Citation > 5,000) with a high level of influence (Figure 5A). To visualize the analysis, journal documents, citation frequency, and total link strength over time were also presented (Figure 5B). Journal of the American College of Cardiology had the highest total link strength and citation, reflecting its importance in publishing studies on the health management of patients undergoing valve replacement. Furthermore, a heatmap was used to depict the annual publication trends of the top 30 productive journals between 2013 and 2022 (Figure 5C). Publications on the health management of patients undergoing valve replacement rapidly increased in more than half of the journals since 2018. Journals publishing these studies had the best publication trend in 2021.

**Table 3:** Top 10 journals and co-cited journals related to the management of patients with valve replacement.

Rank	Journal	Count	IF (2022)	JCR	Co-cited journal	Citation	IF (2022)	JCR
1	Catheterization and Cardiovascular Interventions	133	2.585	Q3	Journal of the American College of Cardiology	10,316	27.203	Q1
2	Journal of Cardiothoracic and Vascular Anesthesia	89	2.894	Q3	Circulation	7,430	39.918	Q1
3	American Journal of Cardiology	84	3.133	Q3	New England Journal of Medicine	5,484	176.079	Q1
4	JACC-cardiovascular Interventions	80	11.075	Q1	European Heart Journal	4,852	35.855	Q1
5	Journal of Cardiac Surgery	62	1.778	Q3	JACC-cardiovascular Interventions	4,586	11.075	Q1
6	Frontiers in Cardiovascular Medicine	61	5.846	Q2	Annals of Thoracic Surgery	4,281	5.102	Q1
7	Journal of Thoracic and Cardiovascular Surgery	58	6.439	Q1	Journal of Thoracic and Cardiovascular Surgery	3,918	6.439	Q1
8	Journal of the American College of Cardiology	57	27.203	Q1	American Journal of Cardiology	3,498	3.133	Q3
9	International Journal of Cardiology	49	4.039	Q2	European Journal of Cardiothoracic Surgery	2,456	4.534	Q1
10	European Journal of Cardiothoracic Surgery	48	4.534	Q1	Catheterization and Cardiovascular Interventions	2,257	2.585	Q3

### 3.5 Analysis of co-cited references and reference bursts

To identify critical literature, the top 10 references cited by articles related to the health management of patients undergoing valve replacement were examined (Table 4). Seven, two, and one references were published in the New England Journal of Medicine (IF: 176.079), the Journal of the American College of Cardiology (IF: 27.203), and the European Heart Journal (IF: 35.855), respectively. The most cited reference was “Transcatheter Aortic-Valve Implantation for Aortic Stenosis in Patients Who Cannot Undergo Surgery” with 597 citations, followed by “Transcatheter vs. Surgical Aortic-Valve Replacement in High-Risk Patients” with 557 citations. Despite the perioperative risks, these two studies suggested that transcatheter aortic valve replacement was a viable alternative for high-risk patients with aortic stenosis and emphasized the importance of informed decision-making and optimal surgical approach (28, 29). The third most cited study was “Transcatheter or Surgical Aortic-Valve Replacement in Intermediate-Risk Patient” with 463 citations. This study evaluated intermediate-risk patients with aortic stenosis and demonstrated that the outcomes of transcatheter aortic valve replacement were comparable to those of surgical aortic valve replacement in terms of the primary endpoint of death or disabling stroke over two years (30).

**Table 4:** Top 10 co-cited references related to the management of patients with valve replacement.

Rank	Title	DOI	First author	Journal	Year	Citations
1	Transcatheter Aortic-Valve Implantation for Aortic Stenosis in Patients Who Cannot Undergo Surgery	10.1056/nejmoa1008232	Leon MB	N Engl J Med	2010	597
2	Transcatheter versus Surgical Aortic-Valve Replacement in High-Risk Patients	10.1056/nejmoa1103510	Smith CR	N Engl J Med	2011	557
3	Transcatheter or Surgical Aortic-Valve Replacement in Intermediate-Risk Patients	10.1056/nejmoa1514616	Leon MB	N Engl J Med	2016	463
4	2017 ESC/EACTS Guidelines for the Management of Valvular Heart Disease	10.1093/eurheartj/ehx391	Baumgartner H	Eur Heart J	2017	393
5	Transcatheter Aortic-Valve Replacement with a Balloon-Expandable Valve in Low-Risk Patients	10.1056/nejmoa1814052	Mack MJ	N Engl J Med	2019	376
6	Transcatheter Aortic-Valve Replacement with a Self-Expanding Valve in Low-Risk Patients	10.1056/nejmoa1816885	Popma JJ	N Engl J Med	2019	298
7	2014 AHA/ACC Guideline for the Management of Patients with Valvular Heart Disease: Executive Summary	10.1016/j.jacc.2014.02.537	Nishimura RA	J Am Coll Cardiol	2014	278
8	Surgical or Transcatheter Aortic-Valve Replacement in Intermediate-Risk Patients	10.1056/nejmoa1700456	Reardon MJ	N Engl J Med	2017	272
9	Transcatheter Aortic-Valve Replacement with a Self-Expanding Prosthesis	10.1056/nejmoa1400590	Adams DH	N Engl J Med	2014	258
10	Updated Standardized Endpoint Definitions for Transcatheter Aortic Valve Implantation the Valve Academic Research Consortium-2 Consensus Document	10.1016/j.jacc.2012.09.001	Kappetein AP	J Am Coll Cardiol	2012	210

N Engl J Med, New England Journal of Medicine; Eur Heart J, European Heart Journal; J Am Coll Cardiol, Journal of the American College of Cardiology.

**Figure 6:**
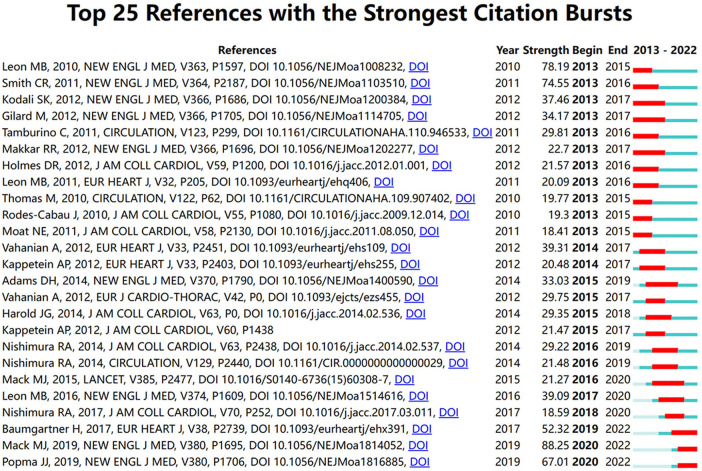
Top 25 references with the strongest citation bursts. Description: “Strength” refers to the degree to which the reference is heavily cited in the short term. “Begin” and “End” indicate the time when the reference burst started and ended, respectively.

To address the concerns about the health management of patients undergoing valve replacement, the top 25 references exhibiting the strongest citation bursts were analyzed and chronologically presented (Figure 6). The focus from 2013 to 2022 was analyzed. Among the studies from 2020 to 2022, the references published by Mack MJ (31) and Popma JJ (32) exhibited increased bursts. Both articles compared the composite endpoint incidence of death and stroke after transcatheter aortic valve replacement and surgical aortic valve replacement at one or two years. The short-term outcomes of transcatheter aortic valve replacement were similar to those of surgical aortic valve replacement in low-risk patients with the added benefit of short recovery time.

### 3.6 Analysis of research hotspots and frontier

The research hotspots and frontiers were analyzed by examining author keywords, which encapsulate the main ideas of the manuscript. Before analysis, the keywords were consolidated to obtain 3,815 author keywords. Table 5 shows the top 10 author keywords with the highest frequency and the author keywords related to management. Among the author keywords, six were linked to aortic valve disease, indicating a significant focus on the health management of patients undergoing aortic valve replacement. The author keywords related to management were divided into the following four categories: anticoagulant management, implant management, complication management, and holistic nursing management. Anticoagulation management-related keywords were frequently used and included “anticoagulation” (72 times) and “warfarin” (39 times). Implant management-related keywords comprised “pacemaker” (62 times), “prosthesis” (56 times), and “bioprosthetic” (49 times). Complication management-related keywords comprised “complication” (67 times), “bleeding” (38 times), and “vascular complications” (35 times). Meanwhile, holistic nursing management-related keywords mainly comprised “quality of life” (24 times) and “frailty” (19 times).

**Table 5:** Top 10 author keywords and author keywords about management content related to the management of patients with valve replacement.

Rank	Author keywords	Occurrences	Total link strength	Author keywords about management content	Occurrences	Total link strength
1	Transcatheter aortic valve replacement	701	2,929	Anticoagulation	72	403
2	Aortic stenosis	671	2,542	Complication	67	243
3	Transcatheter aortic valve implantation	547	1,952	Pacemaker	62	492
4	Aortic valve replacement	334	1,175	Prosthesis	56	317
5	Surgical aortic valve replacement	115	464	Bioprosthetic	49	197
6	Mitral valve replacement	98	410	Warfarin	39	154
7	Valve replacement	88	378	Bleeding	38	184
8	Aortic valve	79	302	Vascular complications	35	145
9	Anticoagulation	72	403	Quality of life	24	308
10	Mitral regurgitation	72	390	Frailty	19	80

**Figure 7:**
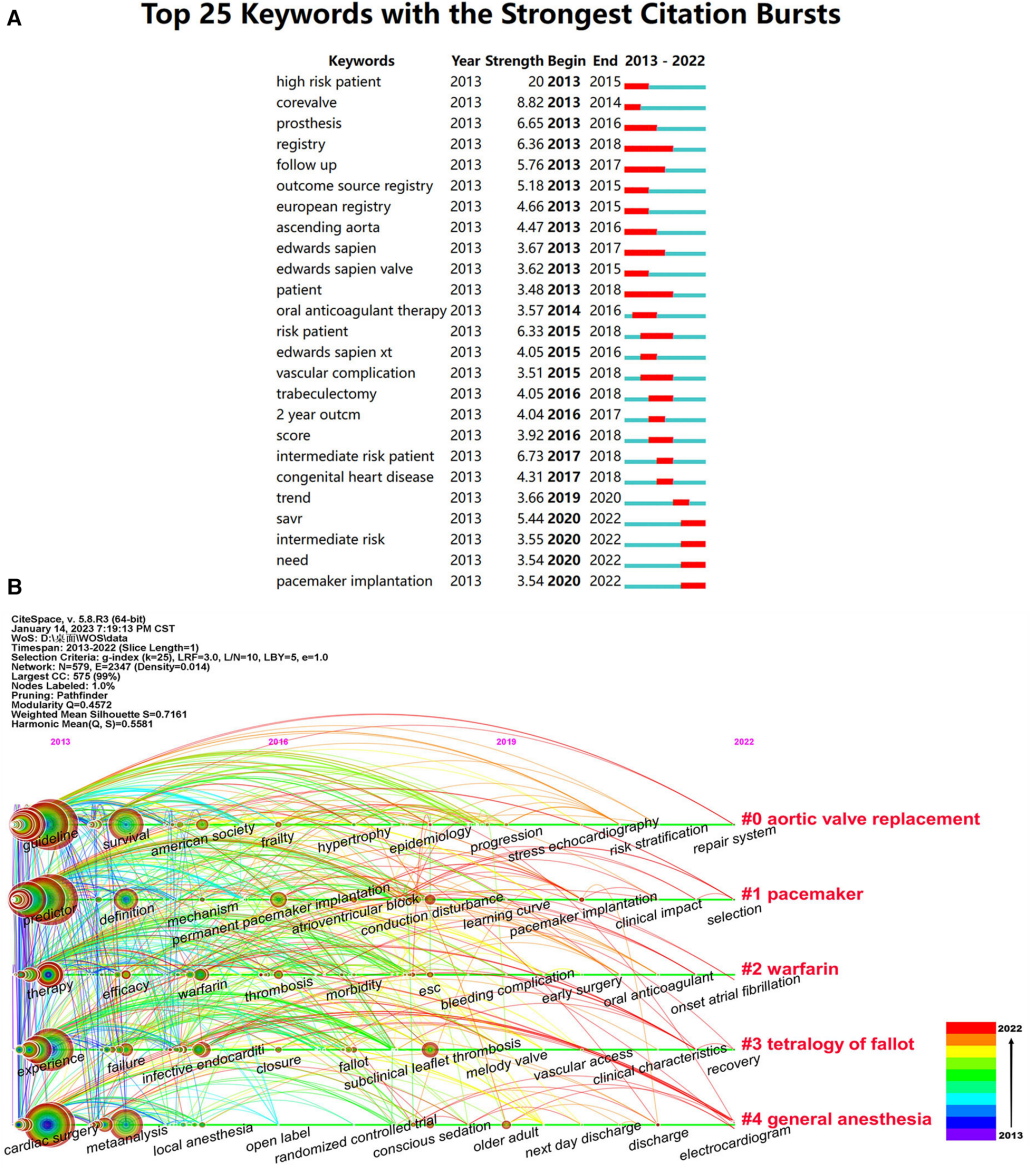
Keywords burst and timeline viewer on the health management for valve replacement patients. (**A**) Top 25 keywords with the strongest citation bursts. Description: “Strength” means the extent to which a keyword receives attention in the short term. “Begin” and “End” indicate the time when the keyword burst started and ended, respectively. (**B**) CiteSpace visualization map of timeline viewer. Description: Each horizontal line represents a cluster, and each node represents a keyword. The node size indicates the keyword occurrence frequency, and the node position is related to the year when the document containing the keyword first appeared.

Keyword burst refers to the frequent usage of keywords and enables the understanding of research forefront, changes in research focus, and the latest trends in research hotspots. The frequency of the keyword usage indicates the strength of the research focus. Figure 7A presents the top 25 keywords exhibiting the strongest citation bursts related to the health management of patients undergoing valve replacement. “High-risk patient” had the strongest citation bursts, followed by “corevalve”, “intermediate risk patient” and “prosthesis”. From a temporal perspective, “high risk patient”, “corevalve” and “prosthesis” emerged earlier and were subjects of early interest. Since 2018, “SAVR”, “intermediate risk”, “need” and “pacemaker implantation” have started to burst and continue until now. These findings revealed that the focus on the management of patients undergoing valve replacement shifted from “high-risk patient” to “intermediate risk.”

CiteSpace can depict a timeline analysis of keywords, explore hot keywords’ rise and fall process, and study the vein of a certain field. The adjustment parameters yielded 12 clusters. Five clusters containing the highest number of keywords are shown in Figure 7B. The largest cluster was labeled “Aortic valve replacement,” suggesting an increased focus on aortic valve replacement. The focus progressively shifted from adverse event management to cardiac rehabilitation management with prevalent keywords, such as “frailty,” “risk stratification,” “recovery,” and “repair system.”

## 4 Discussion

This study used bibliometric methods to analyze the development trends, research status, and hotspots related to the effective health management of patients undergoing valve replacement. The bibliometric methods can promote academic exchange, predict publication directions, and provide medical management decisions. Additionally, bibliometric methods aid researchers in understanding and discovering the research direction and potential clinical utility of a topic and enable clinicians to proactively decide the best management plan. Various bibliometric tools, including CiteSpace, VOSviewer, HistCite, BibExcel, and bibliometric R packages, are available (33–35). VOSviewer and CiteSpace are the most widely used bibliometric tools. Therefore, we combined VOSviewer, which can succinctly display literature relationships, with CiteSpace, which can reveal the evolution process of topics, for knowledge graph analysis to provide more intuitive results.

### 4.1 Global trends and general information

In this study, 2,771 articles on the health management of patients undergoing valve replacement were searched. The increased number of publications indicates an increasing interest in the health management of patients undergoing valve replacement. In particular, more than 300 papers were published annually since 2018 with the highest number of publications in 2021. High-income countries, especially the USA, contributed to studies on the health management of patients undergoing valve replacement. China and Japan ranked among the top 10 countries contributing to publication although their total link strengths were relatively weak, indicating less international cooperation and increased internal collaboration in academic research. To break through these academic barriers, we recommend that Chinese and Japanese researchers strengthen their exchanges with international institutions that have published multiple authoritative publications in this field, such as Mayo Clinic, Columbia University, and Cleveland Clinic. Additionally, research in low-income and middle-income countries should be improved and linked with high-income countries to avoid publication bias and maintain a scientific balance.

In this study, Italy ranked third in terms of publication numbers and total link strength. However, none of the top 10 co-cited authors were affiliated with institutions in Italy, suggesting that the scientific impact of Italian researchers is inadequate and the research quality must improve. Leon MB from Columbia University in the USA was the most co-cited author. Additionally, the publications of Leon MB received several citations, establishing the author as an expert in the health management of patients undergoing valve replacement. Among the academic journals related to the management of patients undergoing valve replacement, the average IF for the top 10 co-cited journals was 31.192, suggesting that articles published in authoritative journals can pique the interest of the researchers and shape the development trends of the field. Journal of American College of Cardiology had the highest number of citations and total link strength, indicating that it is a relatively mainstream and authoritative journal on studies related to the management of patients undergoing valve replacement.

### 4.2 Hotspots and frontiers

#### 4.2.1 Popular research objects

“Aortic valve replacement” was the largest cluster. Six of the top 10 author keywords were related to the aortic valve, indicating increased focus on the health management of patients with aortic valve replacement. Aortic valve stenosis is the most common VHD (36–39). The incidence of aortic valve disease is expected to increase due to the prevalence of risk factors, such as high blood pressure and diabetes. Researchers are actively investigating novel health management approaches to achieve improved therapeutic effects (40, 41). “Transcatheter aortic valve replacement” was the most frequently occurring keyword from 2013 to 2022. Analysis of the frequently cited references and reference bursts revealed that the outcomes of transcatheter aortic valve replacement are comparable to those of surgical aortic valve replacement in patients with different risks of aortic disease. Transcatheter aortic valve replacement has improved the efficacy of aortic valve disease treatment and is the preferred choice for many patients, especially the older patient (42). Scholarly interest is increasing with the increase in the number of transcatheter aortic valve replacements. Therefore, patients undergoing transcatheter aortic valve replacement are currently popular research subjects in the management of patients undergoing valve replacement.

#### 4.2.2 Health management mode combined with the internet and portable equipment

The frequency of anticoagulation-related keywords was the highest in the last decade. Anticoagulation therapy remains a challenging aspect of medical management, especially for patients undergoing valve replacement (43). Currently, researchers have shown interest in developing anticoagulant health management models using the internet and smart devices with a focus on self-management. Huang et al. (14) proposed an anticoagulant management model with a portable coagulator and online medical communication, allowing patients to monitor the international normalized ratio at home and enabling online consultations. Zhu et al. (44) suggested the development of a warfarin anticoagulation follow-up system after valve replacement surgery and compared the internet-based warfarin anticoagulation management model with the traditional warfarin management model. Additionally, mobile applications, such as “Yixing” have been widely used for anticoagulation therapy in patients undergoing valve replacement. Jiang et al. (45) indicated that smart applications are superior to traditional anticoagulant management strategies in improving disease awareness and the fraction of time in therapeutic range. The internet-based anticoagulation management models and portable health devices are promising paradigms that warrant further studies.

#### 4.2.3 Frailty-based cardiac rehabilitation management strategy

Analysis of the timeline of keywords indicated that the health management of patients undergoing valve replacement encompasses not only disease management but also holistic nursing management, such as cardiac rehabilitation. Frailty analysis is a crucial investigation to promote cardiac rehabilitation in patients undergoing valve replacement (46). Wong et al. (47) reported that frailty is an essential factor for predicting mortality and complications among patients undergoing heart surgery, including valve replacement. Early identification and management of frailty are critical measures for mitigating mortality and improving the quality of life of patients (48). Mach et al. (49) investigated the use of fitness trackers to assess frailty in patients undergoing valve replacement and demonstrated that wearable health monitoring devices are as effective as traditional methods and provide reliable and objective data. Additionally, frailty management prioritizes early cardiac rehabilitation programs that combine exercise and rehabilitation to improve the exercise tolerance and functional status of patients undergoing valve replacement (50). Although frailty has been extensively studied, specific prevention and health management strategies have not been developed (51). Therefore, further studies are warranted to develop interventions for frail patients undergoing valve replacement.

### 4.3 Limitations

This novel bibliometric analysis of studies on the management of patients undergoing valve replacement had some limitations. As this study retrieved literature from the WOSCC database, relevant literature published in other databases was not analyzed. Additionally, bibliometrics can provide a comprehensive overview of research progress but cannot assess the quality of individual documents. Furthermore, incomplete keyword extraction by software and the diversity of subject terms may affect the number of keywords. To address this limitation, important keywords were manually consolidated to identify the current hotspots and frontiers in patient management after valve replacement surgery. Despite these limitations, this study provides useful insights into the health management of patients undergoing valve replacement, guides the development of disciplines, and enables clinical applications in this field.

## 5 Conclusion

In this study, the WOSCC database was queried for studies on the health management of patients undergoing valve replacement. Bibliometric analysis and data visualization were performed using CiteSpace and VOSviewer software. The findings of this study indicate that the health management of patients undergoing valve replacement has piqued the interest of the scientific community in the last decade. The country with the highest number of publications and the highest academic influence was the USA. Research hotspot analysis indicated that patients undergoing transcatheter aortic valve replacement are the most popular research subjects. Future studies must focus on the following two main areas: the exploration of internet-based anticoagulant management models and portable health devices and the implementation of cardiac rehabilitation management strategies to reduce frailty in patients after valve replacement. These findings provide valuable insights into the development and future directions of patient health management in the context of valve replacement.

## Data Availability

The raw data supporting the conclusions of this article will be made available by the authors, without undue reservation.
